# Percutaneous cryoablation in soft tissue tumor management: an educational review

**DOI:** 10.1186/s13244-024-01822-5

**Published:** 2024-11-18

**Authors:** Sylvain Bodard, Ruben Geevarghese, Leo Razakamanantsoa, Julien Frandon, Elena N. Petre, Clement Marcelin, François H. Cornelis

**Affiliations:** 1 Department of Radiology, Memorial Sloan Kettering Cancer Center, 1275 York Avenue, New York, NY 10065 USA; 2 Weill Cornell Medical College, 1300 York Avenue, New York, NY 10065 USA; 3 Department of Radiology, Necker Hospital, University of Paris Cité, 149 rue de Sèvre, 75015 Paris, France; 4 Laboratoire d’Imagerie Biomédicale, Sorbonne University, CNRS UMR 7371, INSERM U 1146, 75006 Paris, France; 5 Department of Interventional Radiology and Oncology, Sorbonne University, Tenon Hospital, 4 rue de la Chine, 75020 Paris, France; 6 Radiology Department, Nimes University Hospital, Nimes, France; 7 Department of Radiology, Centre Hospitalo-Universitaire de Bordeaux, 33076 Bordeaux, France

**Keywords:** Ablation techniques, Cryoablation, Soft tissue tumor, Educational review

## Abstract

**Background:**

Percutaneous cryoablation (PCA), having shown effectiveness in treating liver, lung, prostate, breast, and kidney tumors, is now gaining attention for the treatment of soft tissue tumors. PCA functions by freezing tissue, which induces ice crystal formation and cell death without damaging collagen structures. Technical considerations include the selection and handling of cryoprobes and cryogenic agents, procedural duration, and choice of image guidance for precision. This review aims to synthesize the mechanisms, applications, and technical aspects of PCA in the treatment of soft tissue tumors.

**Methods:**

Adhering to PRISMA 2020 guidelines, a review was conducted of studies published prior to March 2024 that investigated PCA of soft tissue tumors. The review focused on technical and procedural aspects of cryoablation, cryobiological principles, cellular and tissue responses to extreme cold, intra- and post-procedure physiological mechanisms during and post-procedure, and main clinical applications.

**Results:**

PCA is efficient in treating soft tissue tumors, including desmoid tumors, vascular malformations, and abdominal wall endometriosis. Several cryobiological mechanisms are involved, notably ice crystal formation, cellular dehydration, osmotic effects, and the inflammatory response, all of which contribute to its efficacy. Key technical aspects include the choice of cryoprobes, cryogenic agents (argon gas or liquid nitrogen), and the duration and control of freezing/thawing cycles. PCA also frequently outperformed traditional treatments like surgery and radiotherapy in terms of pain reduction, tumor size reduction, and patient outcomes. Moreover, its nerve sideration properties make it effective under local anesthesia.

**Conclusion:**

Demonstrating substantial pain reduction, tumor size decrease, and high technical success rates, PCA offers a promising and minimally invasive alternative for soft tissue tumor treatment.

**Critical relevance statement:**

Percutaneous cryoablation provides a minimally invasive, precise alternative for soft tissue tumor management, advancing clinical radiology by offering effective treatment with reduced patient risk and enhanced outcomes through image-guided procedures.

**Key Points:**

- Percutaneous cryoablation (PCA) offers a promising, minimally invasive alternative for managing soft tissue tumors.
- PCA employs image-guided techniques to accurately target and treat tumors, ensuring high precision and control.
- PCA preserves structures like collagen, reduces pain, decreases tumor size, and generally enhances patient outcomes.

**Graphical Abstract:**

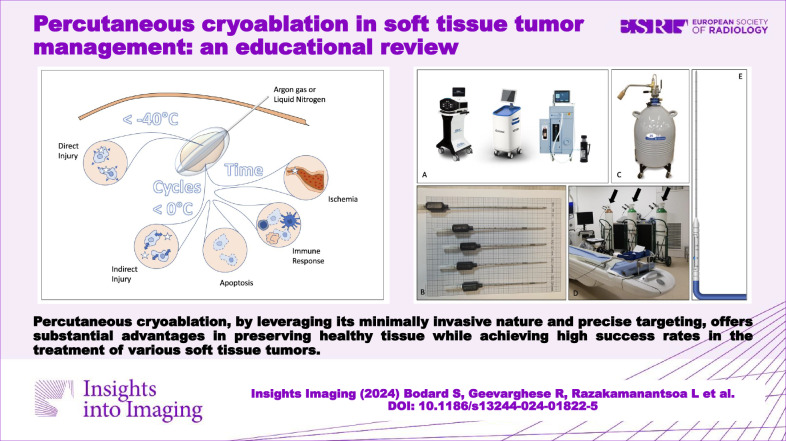

## Introduction

Soft tissue tumors, with an incidence of about 300 cases per 100,000 people [1], exhibit a wide range of complex and diverse histological presentations, including benign lesions such as abdominal wall endometriosis (AWE) and vascular malformations, desmoid tumors, and malignant lesions such as sarcomas [2]. Benign lesions are more common, while malignancies account for less than 1% [3, 4].

Percutaneous image-guided ablation is increasingly used as a substitute for surgical procedures in various cases, especially in patients for whom surgery poses risks or is deemed too complicated [5]. Percutaneous cryoablation (PCA), which involves the image-guided insertion of cryoprobes into tissue, is mainly used for tumors of the liver, lung, prostate, breast, and kidney. Recently, interest has grown in the effectiveness of PCA for the treatment of soft tissue tumors [2, 6]. Unlike radiofrequency ablation (RFA), high-intensity focused ultrasound, or microwave ablation, PCA does not compromise fibrillar structures like collagen. Collagen fibers consist of long, triple-helical protein strands that are less susceptible than cellular water content to the formation of ice crystals during freezing. Furthermore, PCA provides precise control over the ablation zone, and its real-time imaging guidance ensures accurate targeting of tumors, reducing the risk of damage to surrounding healthy tissues. Cold exposure can also slow nerve conduction, reducing nerve activity and dampening pain signals, which produces an anesthetic effect. This improves patient tolerance to the procedure [6–9]. These advantages make PCA a preferred treatment compared to other ablation techniques.

This educational review aimed to provide a comprehensive synthesis of PCA mechanisms for soft tissue tumors, offering an overview of PCA’s primary applications while considering the distinct anatomical and tumoral constitution of soft tissue as well as PCA’s technical and practical considerations.

## Methods

**Fig. 1:**
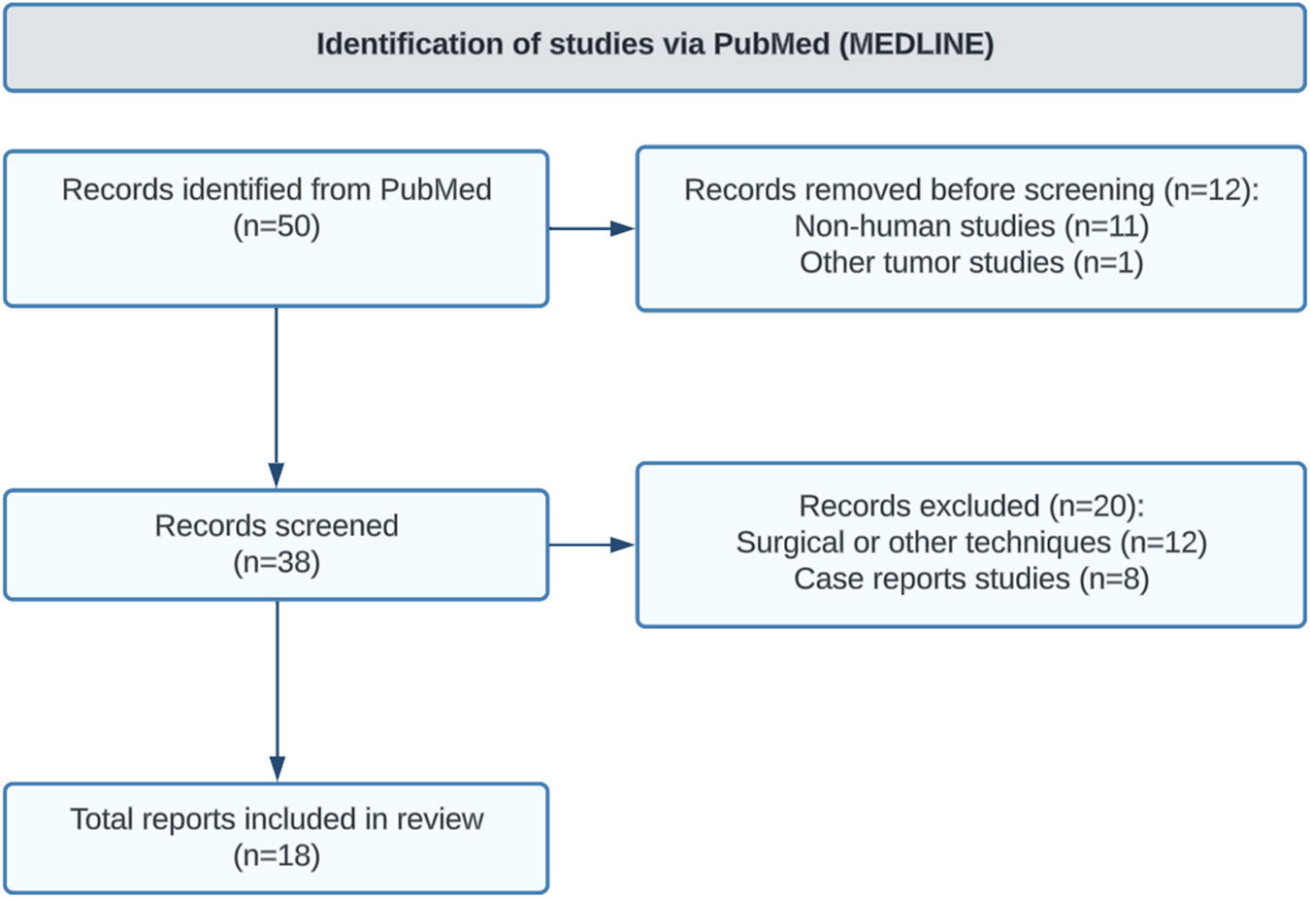
Research methodology

An analysis of studies published prior to March 2024 that focused on PCA of soft tissue tumor was conducted in accordance with the PRISMA 2020 guidelines, with registration in NIHR-PROSPERO (Fig. 1).

### Objectives

This study aimed to assess the technical and procedural aspects of PCA in soft tissue, emphasizing the cryobiological principles underlying its effectiveness. The study included a comprehensive analysis of cellular and tissue responses to extreme cold temperatures, the physiological mechanisms activated intra- and post-procedure, and a review of main clinical applications.

### Methodology

This study detailed the cryoprobes utilized, the stepwise procedural workflow, the types of cryogenic agents used, and the role of imaging guidance in enhancing precision and safety. It also reported the efficacy and adverse effects (AE) of PCA in treating main soft tissue tumors.

### Search strategy

A search of the PubMed (MEDLINE) database was conducted on March 1, 2024, to identify relevant literature using a predefined strategy. This platform was chosen for its accessible and high-quality peer-reviewed articles. Table 1 details the search terms. Two reviewers independently screened each article, including references from pertinent systematic reviews and meta-analyses.

**Table 1:** Search terms

Cryoablation OR Cryotherapy
AND
Technique OR Cryobiology OR Mechanisms OR Technology OR Physiology
AND
Soft tissue tumor OR Desmoid tumor OR Desmoid fibromatosis OR Vascular malformations OR Abdominal wall endometriosis
NOT
Laparoscopic/surgery

### Study selection

Criteria for inclusion comprised studies focusing on PCA mechanisms in soft tissue. Exclusion criteria were studies with only abstracts available, non-English language publications, non-human studies, editorial-style reviews, abstracts and posters, conference papers, and case reports. Based on these criteria, two authors assessed full-text articles. In addition to the parameters above, this study also involved a thorough review of PCA’s primary clinical applications, focusing mainly on desmoid tumors, vascular malformations, and AWE. This review aimed to understand PCA’s practical implications and outcomes in these specific conditions, thereby examining its effectiveness, patient outcomes, and any noted complications or challenges.

### Data extraction

A specifically designed form was used to extract essential information from selected studies. Two radiologists conducted this extraction process independently, with discrepancies resolved through a consensus involving a third senior radiologist. The Quality In Prognosis Studies tool was utilized to control the risk of bias assessment.

## Results

### Cryobiology and application in soft tissue

PCA freezes tissue in order to intracellularly induce ice crystals that cause cell death. Unlike other percutaneous ablation techniques (radiofrequency ablation, high-intensity focused ultrasound, laser, and microwave ablation), PCA preserves fibrillar structures like collagen, without causing damage. Collagen fiber is made up of extended, triple-helix protein strands that are more resistant to ice crystal formation during freezing than cellular water content. Exposure to cold can decrease nerve conduction velocity, which leads to attenuation of pain signals. The resulting anesthetic effect [6, 8, 9] enhances patient comfort during the procedure and enables the execution of PCA using local anesthesia alone. As transient neuropraxia is observed below +5 °C and definitive injuries are observed below −20 °C, the duration of the nerve block depends on various factors, including the temperature reached. Thus, understanding PCA’s effects is crucial for the comprehension of the mechanisms through which freezing temperatures impact cellular and tissue structures in soft tissue. These mechanisms contribute to PCA’s therapeutic outcomes [9, 10]. PCA leads to the following five common effects [6, 8, 9].

#### Direct injury to cells (immediate timing and temperatures below −40 °C)

As PCA temperatures drop below −40 °C, water within the cells begins to freeze and form ice crystals. Ice crystal formation can cause physical damage to cellular structures, including the cell membrane, organelles, and cytoskeleton. The sharp edges of ice crystals can pierce cell membranes, disrupting the integrity of the cells. Changes in membrane permeability can disrupt the balance of ions and molecules within the cell. There is a mismatch between the −40 °C isotherm and the volume of the ice ball, which explains why the cycle must be considered, as well as the extension of margins beyond the boundaries of the lesion [6, 8, 9].

#### Cellular dehydration during freezing, osmotic effects on cells and organelles (immediate timing and temperatures below 0 °C, but correlated to the cycles of freeze/thaw)

Osmotic effects play a significant role in the cellular response to the freezing and thawing process that occurs at temperatures below 0 °C. Osmosis refers to the movement of water across a semipermeable membrane from an area of low solute concentration to an area of high solute concentration. As tissue is exposed to freezing temperatures, water within the cells begins to freeze and form ice crystals. This process results in relative water extraction from the intracellular space, meaning that although the water remains within the cell, it transforms into ice. This transformation effectively removes the water, causing an increase in the concentration of solutes in the unfrozen water and leading to cellular dehydration. Cellular dehydration can contribute to both cell shrinkage and alteration of intracellular structures. The augmentation of the concentration of solutes, creating an osmotic gradient between the intracellular and extracellular spaces, can affect the structural integrity of cell membranes and organelles, leading to a compensatory influx of water into the cell. Multiple cycles will optimize this effect throughout the ice ball, leading to cell death within the ice ball’s whole volume [6, 8, 9].

#### Apoptosis and necrosis (uncontrolled timing and delayed temperatures below 0 °C)

Unplanned cell death (necrosis) occurs, as does the induction of programmed cell death (apoptosis) by compromising the structural integrity and function of cellular organelles, such as mitochondria, endoplasmic reticulum, and nuclei. Mitochondrial damage particularly impacts cellular energy production and contributes to cell death. Apoptosis is triggered by various cellular signals, including those related to osmotic stress and damage to cellular structures [6, 8, 9].

#### Inflammatory response (delayed timing and temperatures below 0 °C)

The release of various signaling molecules and the recruitment of immune cells to the injury site result in delayed inflammation after ice ball melts may last up to 6 months. This inflammatory response contributes to the removal of damaged cells and tissue as part of the healing process. It also contributes to the abscopal effect [11] and may be promoted by adjunction of immune therapies [12].

#### Vascular effects (immediate timing and temperatures below 0 °C, varying in relation to the length of freeze and vessel obstruction, as necrosis can be observed after 20 min, depending on tissue susceptibility to ischemia)

Osmotic effects contribute to vasoconstriction, or the reduction of blood flow to treated tissue. Ice also occludes the vessels with a diameter less than 3 mm, which explains why the intensity of this vascular effect may be related to freeze duration. Both contribute to tissue damage by creating an ischemic environment and then necrosis. Reduced blood flow may also help to minimize intraprocedural bleeding, but does not affect post-procedural bleeding when ice melts. Bleeding risk can be mitigated by avoiding the puncture of large vessels, or by using active thawing at the end of the procedure to avoid cracks in the ice. A slow passive thaw should be prioritized, and the needle should be removed without any active thawing [6, 8, 9].

**Fig. 2:**
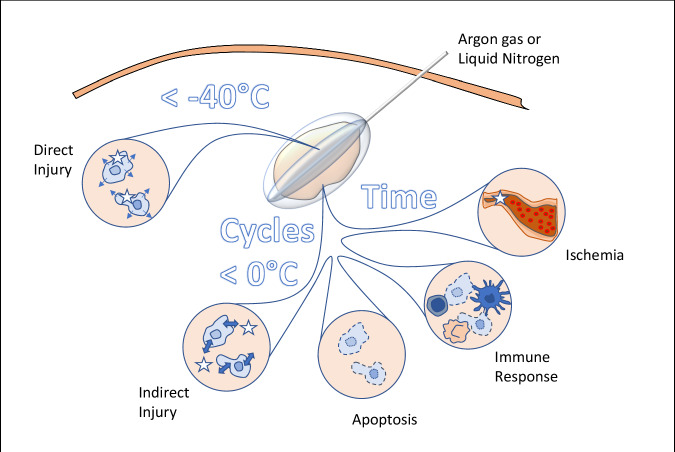
Illustration of the effects of cryoablation

Figure 2 summarizes these five common effects of PCA.

### Technical aspects

#### Cryoprobes

**Fig. 3:**
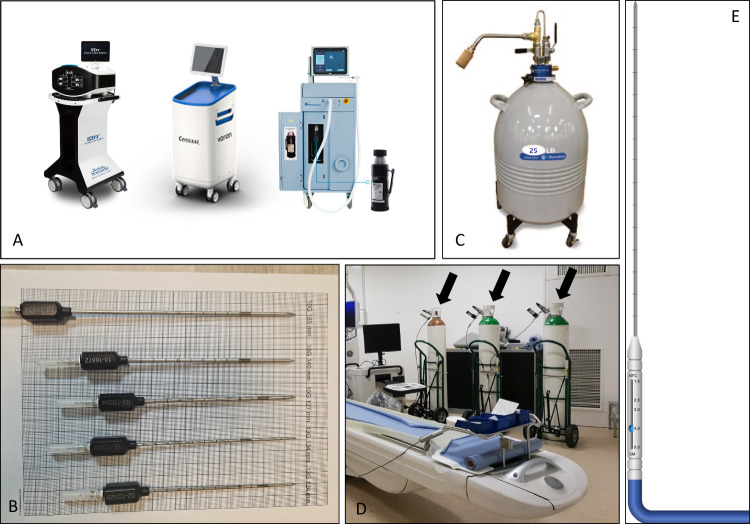
Material used for PCA performance: (**A**) available devices, (**B**, **E**) needles, and (**C**, **D**) cryogenic agents

Cryoablation is typically performed using specialized applicators called cryoprobes. These devices have a chamber that exposes tissue to a cryogenic agent’s freezing temperature, thereby creating an “ice ball” around the probe and ensuring controlled and precise treatment. Cryoprobes are available in gauge sizes from 8 to 17 and in varying chamber lengths that may be advantageously adjusted from 1 to 5 cm. These parameters affect the volume of the ice ball in three dimensions (length × diameter) to obtain ice balls of up to 5–6 cm in diameter and length within soft tissue. Some needles are insulated along the shaft of the needle, while others are not. Thus, operators must carefully warm the skin to avoid thermal injuries. Combination or repositioning of cryoprobes enables both the covering of large lesions and the adjustment of the ice ball to mimic lesion shape. Adequate cryoprobe selection is at the operator’s discretion. The use of larger decompression chambers (10- to 13-gauge cryoprobes) can reduce the number of probes needed per procedure (in turn controlling costs and procedural time), while the use of multiple probes optimizes the ice ball’s ability to conform to lesion shape [6, 8, 9]. Figure 3 shows materials used for PCA performance.

#### Cryoablation procedure

Influencing both procedure efficacy and safety, time is a critical factor in PCA treatment. The freezing cycle’s initial goal is to rapidly cool the tissue and form an ice ball around the probe. The duration of this phase and the type of cryoprobe selected influence both the extent and depth of tissue freezing. The heat-sink effect of soft tissue also plays a significant role. In soft tissue, ice may be observed more quickly than in other organs (except bone). This is particularly true of hypovascular lesions. Operators must carefully monitor ice growth. Once the ice ball is formed, effective tissue ablation requires the maintenance of freezing temperatures for an appropriate duration. Initial recommendations are usually 10 min per cycle, but appropriate freeze duration varies depending on the size and type of the targeted tissue, as well as on the type and number of cryoprobes selected.

**Fig. 4:**
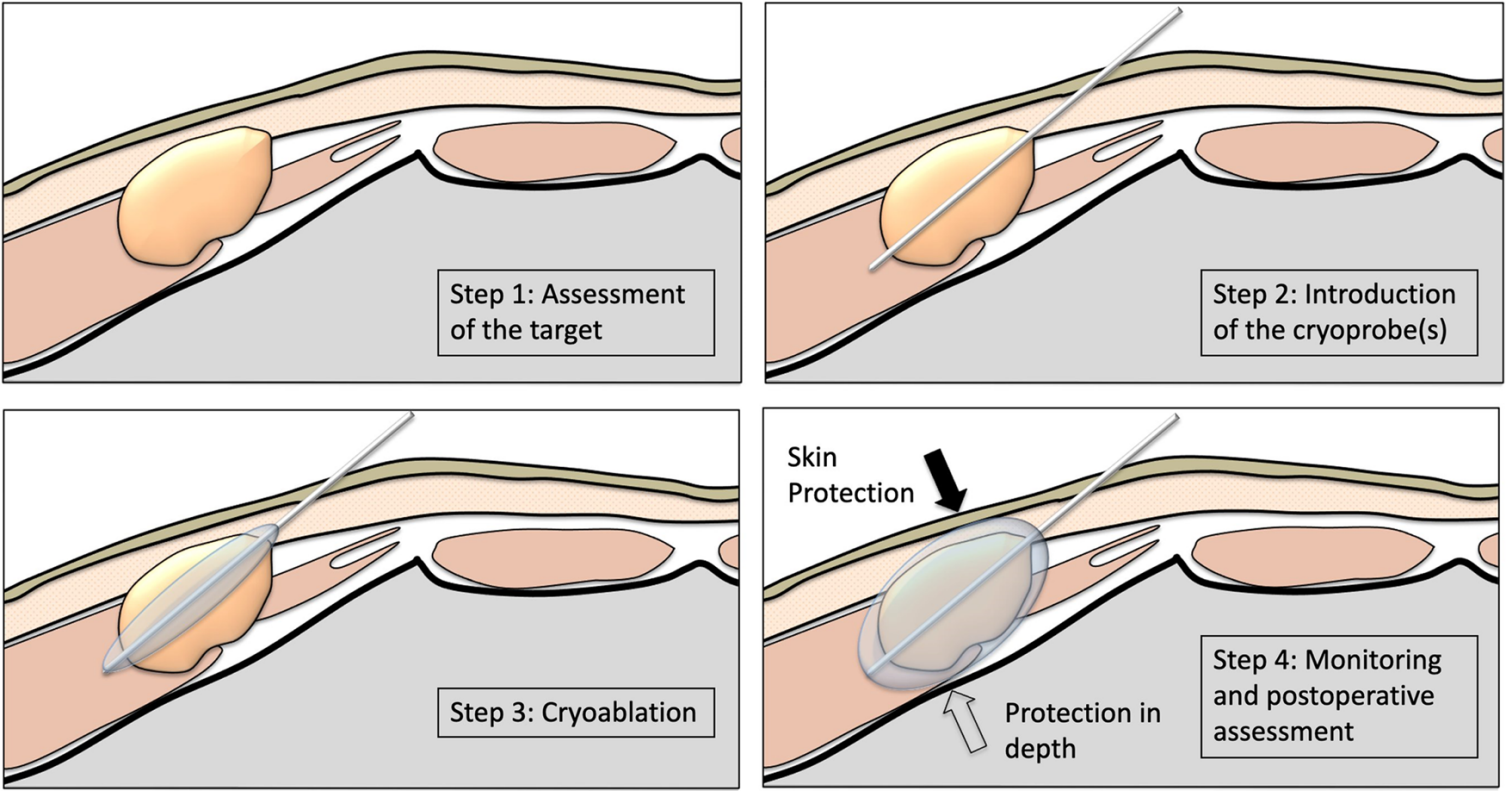
Procedure steps

Ice growth may also be adjusted by decreasing the system’s power when the ice ball completely encompasses the lesion, or by slowing the process to maintain freezing and optimize vascular effects. After the desired initial freezing duration, the thawing phase is initiated. Controlled thawing is as crucial as freezing, and the rate and duration of thawing also impact the procedure’s success. Rapid thawing may lead to uneven effects, such as ice cracks, while slow thawing may hypothetically result in incomplete tissue ablation as osmotic effects will be reduced. PCA often involves at least two full freeze/thaw cycles to enhance the effectiveness of tissue destruction by mainly increasing dehydration and ischemia. Although the number and duration of cycles depend on the specific goals of the procedure, optimizing these parameters is crucial for the achievement of the desired therapeutic effects in soft tissue while minimizing damage to surrounding healthy tissue, such as nerves and skin [6, 8, 9]. Figure 4 shows procedural steps.

#### Cryogenic agents

The choice of cryogenic agent—whether argon gas or liquid nitrogen—plays a significant role in the effectiveness and safety of PCA procedures. Each cryogenic agent has unique properties that enable it to absorb heat from its surroundings and induce freezing. Cryoprobes deliver these agents to the target tissue. No gas or nitrogen should be released around the needle, and the operators must check each needle’s integrity before the procedure [6, 9]. Table 2 summarizes the routinely used cryogenic agents.

**Table 2:** Routinely used cryogenic agents

	Boiling point (°C)	Inversion temperature at 1 atm (Kelvin)	Operational pressure of the system (PSI)	Advantages	Limitations
**Argon gas**	−185.8	794	4000	Inert and does not support combustion	High-pressure tank needed; cost
**Helium gas**	−268.9	45	4000	Inert and does not support combustion	High-pressure tank needed; cost; can be replaced by cauterization
**Liquid nitrogen**	−196	621	100	Readily available and cost-effective; low operational pressure (100 PSI); no tank needed	Protection measure; time needed to produce heat for extraction

*PSI* pounds per square inch

Argon gas expands and cools upon release in the decompression chamber and returns to the system. The Joule–Thomson effect describes the phenomenon observed in thermodynamics when a gas undergoes a throttling process, which involves a sudden fluid expansion via a valve or porous plug. This effect is consistent with the conservation of energy, which is a fundamental law in physics that states that the total energy of an isolated system remains constant over time and involves the interplay between kinetic and potential energy. The Joule–Thomson process is an isenthalpic process that occurs at constant enthalpy. Enthalpy is a thermodynamic property that includes internal energy and the product of pressure and volume. PCA systems using argon gas require high-pressure tanks (4000 pounds per square inch [PSI] argon gas) to maximize the Joule–Thomson effect. The temperature of the gas can change during the throttling process. Depending on the fluid’s initial conditions (pressure and temperature), the Joule–Thomson coefficient may be either positive or negative. The inversion temperature is the specific initial temperature and pressure at which the Joule–Thomson coefficient is zero. The effect is positive above the inversion temperature, and negative below the inversion temperature. If positive, the temperature of the fluid increases during the throttling process, absorbing the tissues’ heat and then producing an ice ball, as observed with argon gas. If negative, the temperature of the fluid decreases as it undergoes throttling and releases heat, as observed with helium gas. This explains why helium is used to thaw in some systems, but can be replaced by cauterization [8, 9, 13].

Allowing liquid nitrogen to flow from the system to the cryoprobe tip by generating pressure within the system (no external pressure) is another technique for the creation of freezing temperatures. The pressure in the system depends on the cryoprobe and the time of the freezing steps, but the maximum working pressure remains approximately 100 PSI. A thinner cryoprobe requires higher pressure and vice versa. The pressure is highest at the beginning of each freezing step before slowly decreasing. After circulating in the needle, the liquid nitrogen turns to gas at a heat exchange area, and then the gas is heated to be used for the extraction phase if needed [8, 9, 13]. Figure 3 shows cryogenic agents used for PCA performance.

#### Image guidance

Image guidance is a crucial aspect of PCA procedures that ensures both precise and accurate targeting of soft tissue for treatment. Diagnostic imaging modalities like computed tomography, magnetic resonance imaging (MRI), or ultrasonography are often used before the PCA procedure to identify and characterize soft tissue abnormalities, such as tumors or lesions. These images provide detailed anatomical information for planning the optimal PCA approach [9]. The choice of imaging modality depends on various factors, including the location and characteristics of the tumor. For example, ultrasound is often preferred for real-time imaging and guidance due to its ease of use and lack of ionizing radiation. However, CT or MRI might be preferred for deeper or more complex lesions, where the ability to visualize surrounding structures is critical. It is important to note that MRI requires the use of MRI-compatible equipment, which can limit its applicability. Image guidance can also involve combining different imaging modalities to improve accuracy and results. For example, ultrasound, which allows real-time visualization, is frequently combined with CT or MRI to improve tumor visibility and ensure precise targeting during ablation procedures. Studies have shown that these fusion imaging techniques significantly improve the therapeutic efficacy and safety of percutaneous ablation, particularly for tumors that are difficult to visualize with a single modality [14–17].

Real-time imaging is essential to guide the accurate positioning of cryoprobes during the PCA procedure. Computed tomography, MRI, or ultrasonography enable visualization of ice ball formation, with operators using this feedback to determine when a sufficient volume of tissue has been treated and the entire tumor or lesion has been adequately ablated. Continuous monitoring of temperatures within the targeted tissue and surrounding areas is also critical for PCA success [6, 9]. Thermocouples or temperature-sensitive imaging techniques can be employed to ensure that the desired freezing temperatures are achieved while avoiding excessive cooling that may lead to unintended damage. Protective measures, such as the injection of saline or CO_2_ or the application of gels, shields, or insulating pads, may be employed to shield healthy structures like the skin from direct exposure to extreme cold. These protective barriers act as insulators and contribute to the preservation of tissue [9, 18, 19]. Image guidance is sometimes integrated with navigation systems or robotics to visualize the targeted soft tissue in three dimensions [20]. Three-dimensional visualization supports precise navigation of the PCA probe and allows for better spatial awareness during the procedure. After PCA, follow-up imaging, often with MRI, is performed to evaluate treatment success and assess the extent of tissue damage [21, 22]. This final step helps clinicians to confirm the procedure’s effectiveness and to monitor for any potential complications or recurrence of the treated condition.

#### Examples of applications

Bodard et al [2] evaluated 393 PCA procedures for desmoid tumors, including one non-randomized, multicentric trial and several comparisons of PCA to other treatments. Their findings revealed a pain reduction of 79 ± 17% (57–100%), a size reduction of 71.5 ± 9.8% (44–97%), and a good response (complete: 26.33%; partial: 48.75%; stable disease: 29.4%). The overall objective response rate was recorded at 80%. Additionally, the study noted progression-free survival rates between 85.6% (1 year) and 62.5% (2 years), consistent with the literature and similar to surgical results. However, reported response rates and disease control rates were shown to be 68% and 96%, respectively, for radiotherapy; 31% and 67%, respectively, for tamoxifen; and 53% and 80%, respectively, for chemotherapy [2]. Regarding AEs, the authors reported minor complications in 19.88% of patients, such as edema or temporary post-procedural pain increase. Bodard et al reported 4.85% incidence of major AEs, such as skin necrosis or infection, which is lower than that recorded after surgery (about 6%), radiotherapy (37%), or systemic therapies [2].

Moreover, the systematic review of Bodard et al [2] encompassed 58 PCA sessions of vascular malformations, including a prospective trial. They reported a technical success rate of 100% and a pain decrease of 72 ± 25% (63–85%) with a drop from 3–10 to 0–3 (*p* < 0.01) on the Numerical Rating Scale. The reduction in lesion volume was 85% (76–93%), with over 90% complete response at 6 months. This was comparable to sclerotherapy and radiofrequency ablation, which show complete response in 71–100% and 62–100% of cases, respectively [2]. PCA’s effectiveness in targeting both venous malformations and the adjacent tissues has positioned it as the preferred initial treatment in cases where tissue involvement is significant [23]. This is despite PCA having a higher incidence of AEs (78.6%) compared to sclerotherapy (34%, with 16% being severe [24]). In fact, AEs can be managed through short-term steroid medication, dissection, or the maintenance of a minimum of 5 mm from the skin and 3 mm from nerves during PCA application [25].

Finally, assessing 103 PCA sessions that targeted AWE, Bodard et al [2] observed a decrease in pain levels of 82 ± 13% (62–100% and a reduction in visual analog scale scores from 5–9 to 0–5). Hormonal therapy and surgery are the main treatment options for AWE [26]. However, these treatments’ efficacy is often limited in more advanced stages, and they tend to provide only temporary symptom alleviation [27, 28]. Further, surgical treatment requires precise excision of endometriotic lesions while ensuring clear margins and no disruption or dispersal of tiny endometrial tissue fragments. This challenge is further complicated by the disease’s extensively distributed nature and leads to an AWE postoperative recurrence rate of about 4.3% [29]. Most studies reported low AEs, with one exception noting a 2% incidence of severe AE [2], such as bleeding, infection, nerve damage, mesh displacement, or hernia development. In contrast, surgery shows a higher AE rate of about 23% [29]. Hormonal therapy entails various AEs after prolonged use, including irregular bleeding, breast soreness, nausea, bloating, weight gain, hair loss, headaches, depression, anxiety, decrease in bone density, and osteoporosis [27, 28].

These data resulting from the included studies are primarily small-scale, single-site cohorts with small follow-up durations. Comprehensive controlled trials with longer follow-up periods are needed to effectively determine the best treatment approaches for soft tissue tumors. However, the infrequent occurrence of soft tissue tumors poses challenges for establishing and recruiting participants for such trials, which causes treatment methods to remain specific to particular institutions.

## Discussion

PCA distinguishes itself from other ablation methods by its ability to preserve collagen structures while inducing cell death through ice crystal formation. This technique’s effectiveness is enhanced by cryoprobes that enable precise control over ice formation and accurate targeting of lesions. The choice of cryogenic agent is pivotal, with agents like argon gas or liquid nitrogen presenting unique properties that contribute to the freezing process.

Procedural success hinges on real-time imaging technologies like computed tomography, MRI, or ultrasonography, which guide the placement of cryoprobes and monitor ice ball formation. Temperature-sensitive techniques and protective measures permit the preservation of surrounding healthy tissues. Furthermore, PCA’s anesthetic effects—notably its ability to dampen nerve activity and pain signals—enhance patient tolerance. The flexibility in probe selection and ability to optimize the freeze/thaw cycle allow for a tailored approach to each patient’s needs.

PCA’s combination of precision and control makes it a promising tool for the treatment of soft tissue tumors. Bodard et al’s [2] systematic review of 393 PCA procedures for desmoid tumor treatment revealed significant pain and tumor size reductions, with an overall objective response rate of 80%. PCA’s performance was equal to or superior to traditional treatments like surgery, radiotherapy, and chemotherapy. The same review reported a 100% success rate for PCA treatment of vascular malformations, with substantial pain reduction and improvements in quality-of-life indicators. The review also included an analysis of 103 PCA sessions of AWE treatment, revealing a high rate of pain reduction and efficacy, as well as a recurrence rate significantly lower than surgery. While surgical treatments for AWE present risks like severe AEs and aesthetic concerns, PCA could maintain a profile of minimal AEs. PCA has also expanded its scope to include other soft tissue tumors, such as sarcomas [9], retroperitoneal metastases, pelvic sidewall disease, and gynecologic lesions, without the risks linked to surgery or systemic therapies [30]. While interest in PCA use for a range of soft tissue tumors is increasing, the current body of research comprises mainly smaller single studies. A more comprehensive scope of research is required to fully evaluate the efficacy and safety of PCA in these diverse applications. Further, consideration of recent economic factors, such as the increased cost of helium and argon—both critical components of cryoablation procedures—is essential. Similarly, rising expenses may have significant implications for the overall accessibility and affordability of cryoablation therapies in diverse healthcare settings.

In conclusion, PCA’s ability to target lesions with minimal damage to surrounding tissues while offering advantageous anesthetic properties significantly enhances treatment outcomes. Indeed, PCA demonstrates a good success rate and patient tolerance in soft tumor treatment. Nevertheless, and despite its promising results in current studies, there remains a need for broader research to fully ascertain the efficacy and safety of PCA across a wider range of applications.

## Data Availability

Data is available from the authors upon reasonable request.

## Abbreviations

AE: Adverse effect
AWE: Abdominal wall endometriosis
MRI: Magnetic resonance imaging
PCA: Percutaneous cryoablation
PSI: Pounds per square inch
